# Supplementary material for: Synovial microenvironment-influenced mast cells promote the progression of rheumatoid arthritis
Source: Nat Commun. 2024 Jan 2;15:113. doi: 10.1038/s41467-023-44304-w (PMC10761862; doi:10.1038/s41467-023-44304-w)

Supplementary Information for

Synovial microenvironment-influenced mast cells promote the progression of rheumatoid arthritis

Supplementary Table 1. Clinical characteristics of patients with OA and RA

	OA（n=10）	RA (n=11)
Age (years)	70.1±3.35	64.74±9.96
Sex (n; %)		
Male	1 (10%)	1 (9.09%)
Female	9 (90%)	10 (90.91%)
Disease duration (years)	3.2±2.44	3.46±2.91
RF (IU/ml))	9.67±3.32	89.19±115.6
ACPA (RU/ml)	0.62±0.49	84.68±94.54
ESR (mm/h)	30.70±23.96	49.55±25.91
CRP (mg/L)	6.35±3.3	10.32±6.35
Treatment (n, %)		
NSAIDs	n.a.	(1, 9.09%)
GC	n.a.	(1, 9.09%)
MTX+NSAIDs	n.a.	(2, 18.18%)
MTX+ GC	n.a.	(1, 9.09%)
MTX+DMARDs	n.a.	(6, 54.55%)

Average values with standard deviation (±SD) are presented.

RF, rheumatoid factor; ACPA, anti-citrullinated protein antibodies; ESR, erythrocyte sedimentation rate; CRP, C-reactive protein; Mtx, methotrexate; GC, glucocorticoids; NSAIDs, non-steroidal anti-inflammatory drugs; DMARDs, disease modifying anti rheumatic drugs; n.a., not applicable.

Supplementary Table 2. Primer sequences

Human-*CCL2*-F	5'- CAGCCAGATGCAATCAATGCC -3'
Human-*CCL2*-R	5'- TGGAATCCTGAACCCACTTCT -3'
Human-*CCL3*-F	5'- TGATGCAGAGAACTGGTTGC -3'
Human-*CCL3*-R	5'- CAGTGGTCAGTCCTTTCTTGG -3'
Human-*CCL4*-F	5'- AACAGTGACAGTGGACCATCC -3'
Human-*CCL4*-R	5'- TCCATACTCAGGACTCCTCTCC -3'
Human-*CXCL8*-F	5'- ACTGAGAGTGATTGAGAGTGGAC -3'
Human-*CXCL8*-R	5'- AACCCTCTGCACCCAGTTTTC -3'
Human-*IL6*-F	5'- ACTCACCTCTTCAGAACGAATTG -3'
Human-*IL6*-R	5'- CCATCTTTGGAAGGTTCAGGTTG -3'
Human-*TNF*-F	5'- AAGCCTGTAGCCCATGTTGT -3'
Human-*TNF*-R	5'- CAGATAGATGGGCTCATACC -3'
Human-*TUBB*-F	5'- AAGATCCGAGAAGAATACCCTGA -3'
Human-*TUBB*-R	5'- CTACCAACTGATGGACGGAGA -3'
Human-*IL4*-F	5'- ATGGGTCTCACCTCCCAACT -3'
Human-*IL4*-R	5'- GATGTCTGTTACGGTCAACTCG -3'
Human-*IL10*-F	5'- TCAAGGCGCATGTGAACTCC -3'
Human-*IL10*-R	5'- GATGTCAAACTCACTCATGGCT -3'
Human-*IL13*-F	5'- GAGGATGCTGAGCGGATTCTG -3'
Human-*IL13*-R	5'- CACCTCGATTTTGGTGTCTCG -3'

Human-*TGFB1*-F	5'- CAATTCCTGGCGATACCTCAG -3'
Human-*TGFB1*-R	5'- GCACAACTCCGGTGACATCAA -3'

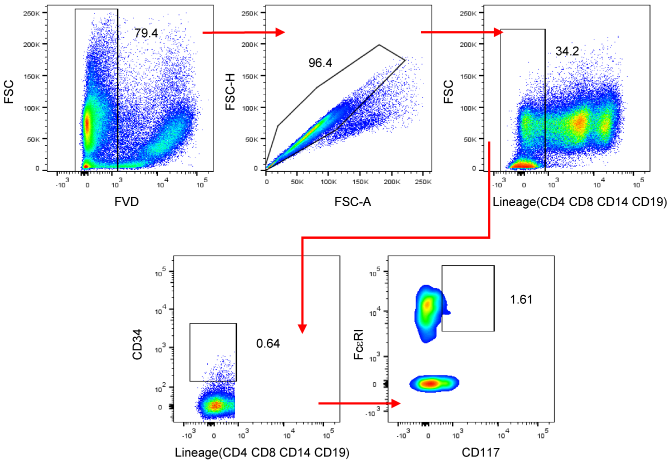

Supplementary Fig. 1 Gating strategy for identifying mast cell progenitors in peripheral blood. MCP were gated as $Lin^{-}CD34^{hi}CD117^{int/hi}Fc\varepsilon RI^{+}$ cells.

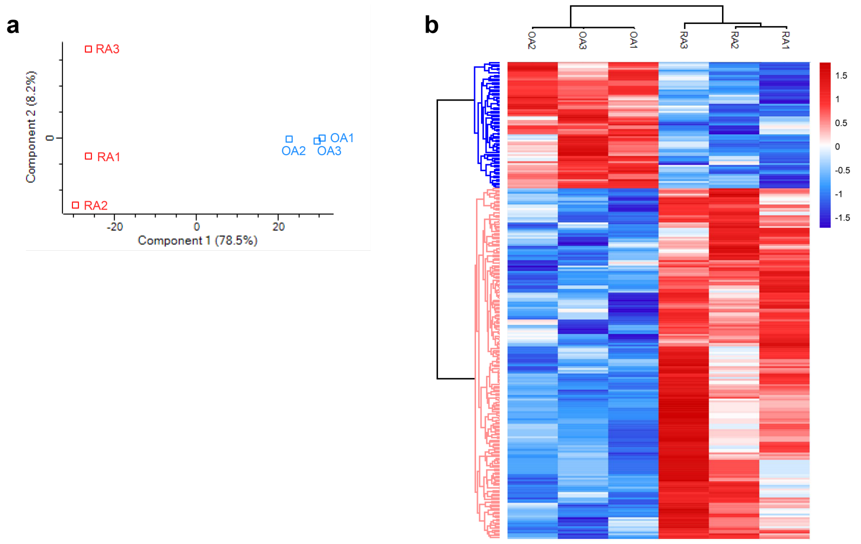

Supplementary Fig. 2 Proteomic analysis of synovial mast cells. (**a**) Principal component analysis (PCA) plot of synovial mast cells from patients with OA and RA. (**b**) Heatmap of all 265 differentially expressed proteins between synovial mast cells from OA and RA patients by hierarchical clustering after z-score normalization. Upregulated and downregulated proteins in RA synovial mast cells were depicted in red and blue, respectively.

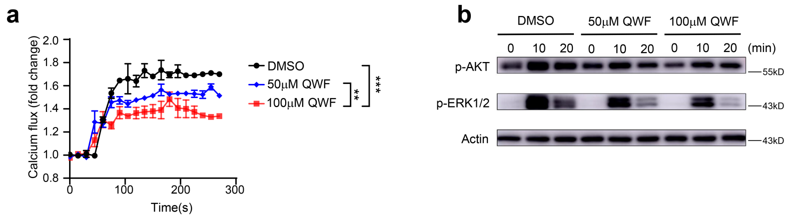

Supplementary Fig. 3 QWF inhibited the activation of mast cells upon RASF stimulation. LAD2 cells were pre-treated with different concentrations of QWF for 30min before stimulated with RASF. Dimethyl sulfoxide (DMSO) was used for control. (**a**) Statistical analysis of intracellular calcium flux of LAD2 cells after RASF stimulation (n=2 for DMSO, n=3 for 50µM QWF and 100µM QWF, pooled from three independent experiments, *P*=0.0002 DMSO vs 100µM QWF, *P*=0.0044 50µM QWF vs 100µM QWF). (**b**) Phosphorylated levels of signaling pathways in LAD2 cells treated with QWF before RASF stimulation. Data are representative of three independent experiments. Data are presented as the mean ± SEM and analyzed using two-way ANOVA (**a**). $^{**}P < 0.01$, $^{***}P < 0.001$. Source data are provided as a Source Data file.

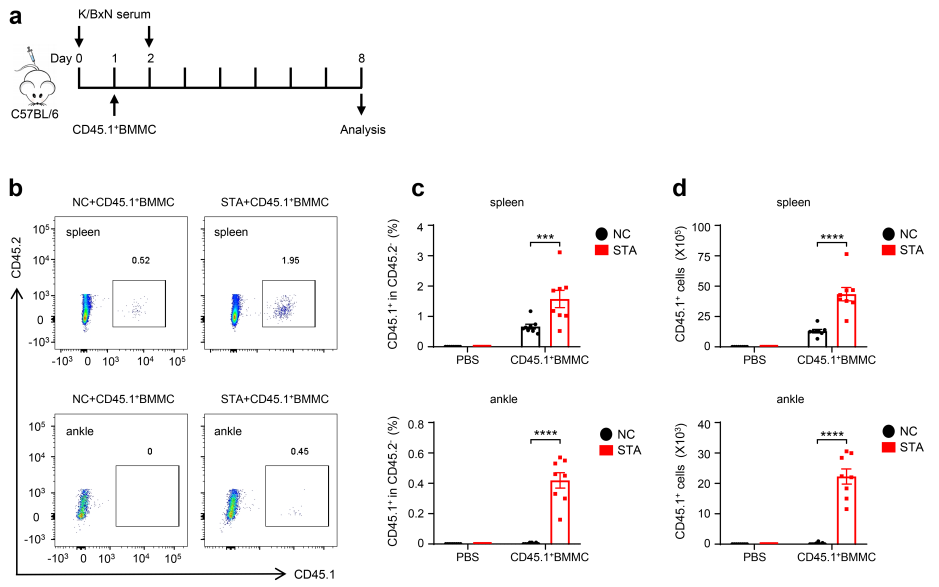

Supplementary Fig. 4 Adoptive transfer of bone marrow derived mast cells (BMMC) in serum transfer induced arthritis (STA) mice. (**a**) The schematic diagram of study design: C57BL/6 mice received two intraperitoneal injections of 150μl K/BxN serum on day 0 and day 2, and 5×10^6 mast cells were adoptively transferred through tail vein injection on day 1. Spleen and ankle joints were harvested on day 8. (**b**) Representative flow plots of CD45.1$^+$cells in the spleen and ankle joints. (**c**) Statistical analysis of frequencies of CD45.1$^+$cells in the spleen and ankle joints (n=8 mice for each group, pooled from two independent experiments, P=0.003 for spleen, P<0.0001 for ankle). (**d**) Statistical analysis of absolute numbers of CD45.1$^+$cells in the spleen and ankle joints (n=8 mice for each group, pooled from two independent experiments, P<0.0001 for spleen and ankle). Data are presented as the mean ± SEM and analyzed using two-way ANOVA (**c**). ***P < 0.001, ****P < 0.0001. Source data are provided as a Source Data file.

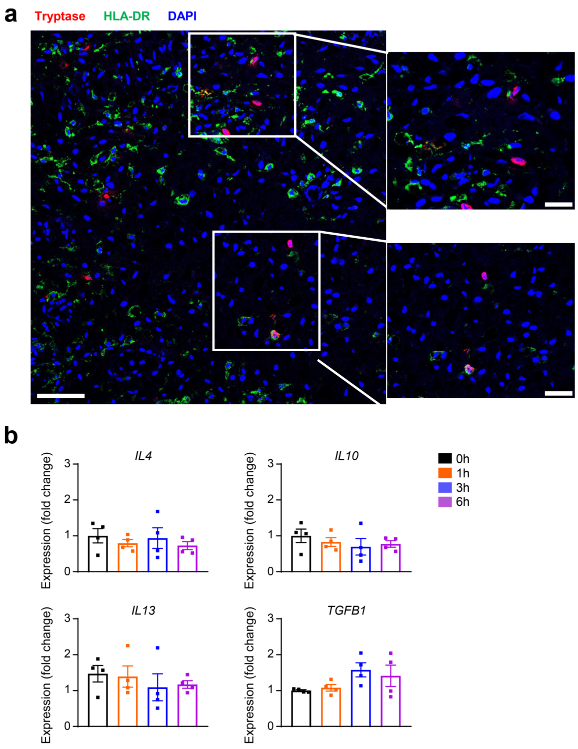

Supplementary Fig. 5 Mast cell phenotype in the RA synovial microenvironment. (**a**) Representative immunofluorescence images of synovial tissues from RA patients. Red, tryptase; Green, HLA-DR; Blue, DAPI. Scale bar: 75μm. The area in the white box is enlarged as an inset. Data are representative of 10 biologically independent samples. (**b**) Cytokine gene expression of LAD2 cells stimulated with 20% RASF for different time intervals were analyzed by qPCR (n=4 biologically independent samples for each group). Data are presented as the mean ± SEM and analyzed using Kruskal-Wallis test (**b**). Source data are provided as a Source Data file.

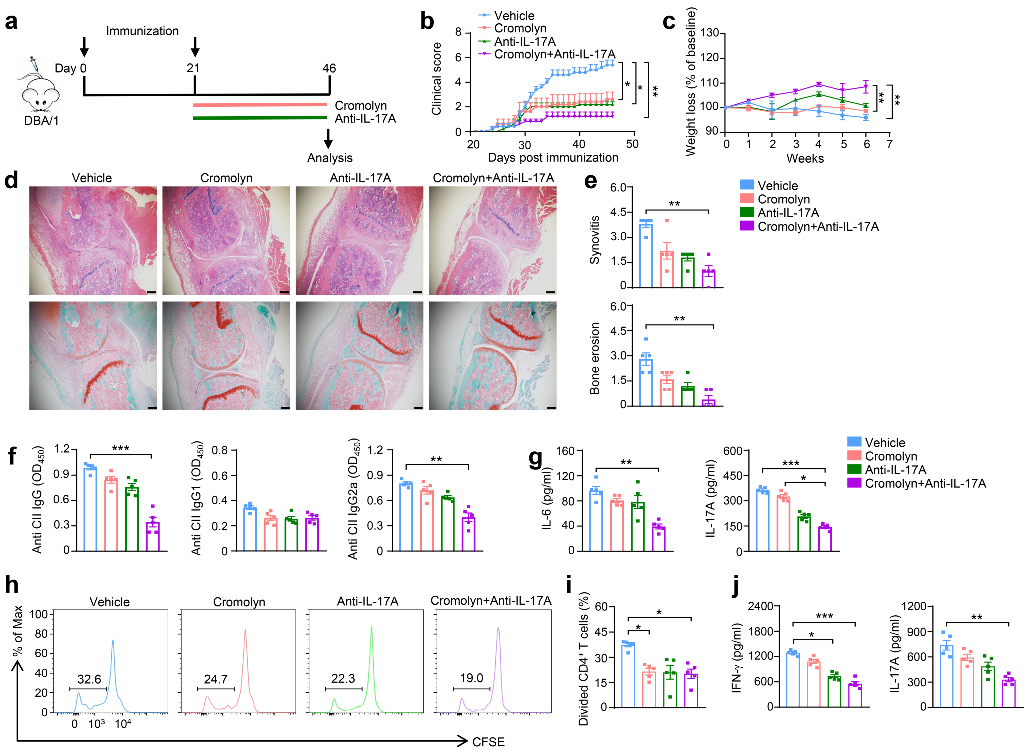

Supplementary Fig. 6 Therapeutic applications of cromolyn sodium and anti-IL-17A attenuated CIA. (**a**) All mice received initial immunization on day 0 and booster immunization on day 21. After being randomly divided into 4 groups, mice were then treated through intraperitoneal injection with the vehicle, cromolyn (25 mg/kg), IL-17A antibody (150 μg each time), or the combination of the cromolyn and IL-17A antibody. Cromolyn and anti-IL-17A treatment were started from day 21, and were both given every other day. (**b**) Arthritis scores were measured every day after booster immunization (n=5 mice for each group, *P*=0.0447 Vehicle vs Cromolyn, *P*=0.0215 Vehicle vs Anti-IL-17A, *P*=0.0016 Vehicle vs Cromolyn+Anti-IL-17A). (**c**) Weight loss of each group showed as a percentage change observed once a week (n=5 mice for each group, *P*=0.0015 Vehicle vs Cromolyn+Anti-IL-17A, *P*=0.0026 Cromolyn vs Cromolyn+Anti-IL-17A). (**d**) Representative images of H&E and Safranin O-fast green

staining of knee joints. Scale bar: 200 μm. (**e**) Statistical analysis of synovitis and bone erosion (n=5 mice for each group, *P*=0.0022 for synovitis, *P*=0.0016 for bone erosion). (**f**) Serum levels of anti-CII IgG, IgG1, and IgG2a were quantified by ELISA (n=5 mice for each group, *P*=0.0005 for IgG, *P*=0.0011 for IgG2a). (**g**) Serum levels of IL-6 and IL-17A were determined by ELISA (n=5 mice for each group, *P*=0.0028 for IL-6, ****P*=0.0006 and **P*=0.0327 for IL-17A). (**h**) Representative flow plots of CFSE-labeled splenocytes gated on $CD3^+CD4^+$ cells after stimulated with CII for 4 days. (**i**) Statistical analysis of proliferation rate after stimulation (n=5 mice for each group, *P*=0.0385 Vehicle vs Cromolyn, *P*=0.0327 Vehicle vs Cromolyn+Anti-IL-17A). (**j**) Inflammatory cytokine release from splenocytes after being stimulated with CII (n=5 mice for each group, **P*=0.0327 and ****P*=0.009 for IFN-γ, *P*=0.0017 for IL-17A). Data are representative of two independent experiments (**b-j**). Data are presented as the mean ± SEM and analyzed using two-way ANOVA (**b**, **c**), Kruskal-Wallis test (**e-g**, **i**, and **j**). *P < 0.05, **P < 0.01, and ***P < 0.001. Source data are provided as a Source Data file.

Uncropped gels for Western blots in Supplementary Fig. 3b

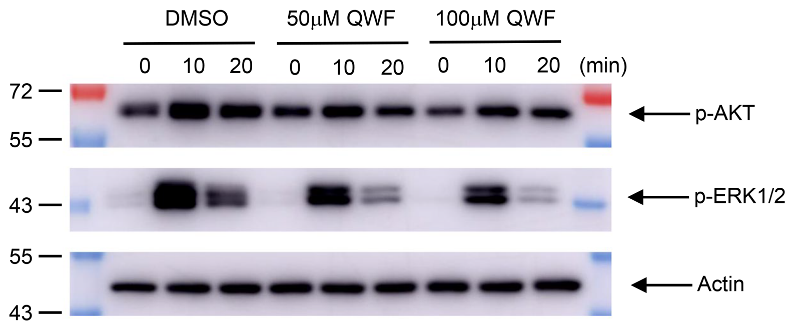

Supplement: Supplementary file 1 — Supplementary Information [file 41467_2023_44304_MOESM1_ESM.pdf]
