# Supplementary material for: Synovial microenvironment-influenced mast cells promote the progression of rheumatoid arthritis
Source: Nat Commun. 2024 Jan 2;15:113. doi: 10.1038/s41467-023-44304-w (PMC10761862; doi:10.1038/s41467-023-44304-w)
Supplement: Supplementary file 3 — Reporting Summary [file 41467_2023_44304_MOESM3_ESM.pdf]

nature portfolio

Corresponding author(s): Xiaochun Peng, Zhaojun Wang, and Guangjie Chen

Last updated by author(s): Nov 25, 2023

Reporting Summary

Nature Portfolio wishes to improve the reproducibility of the work that we publish. This form provides structure for consistency and transparency in reporting. For further information on Nature Portfolio policies, see our Editorial Policies and the Editorial Policy Checklist.

Statistics

For all statistical analyses, confirm that the following items are present in the figure legend, table legend, main text, or Methods section.

n/a	Confirmed	
[ ]	[x]	The exact sample size (*n*) for each experimental group/condition, given as a discrete number and unit of measurement
[ ]	[x]	A statement on whether measurements were taken from distinct samples or whether the same sample was measured repeatedly
[ ]	[x]	The statistical test(s) used AND whether they are one- or two-sided<br>*Only common tests should be described solely by name; describe more complex techniques in the Methods section.*
[x]	[ ]	A description of all covariates tested
[ ]	[x]	A description of any assumptions or corrections, such as tests of normality and adjustment for multiple comparisons
[ ]	[x]	A full description of the statistical parameters including central tendency (e.g. means) or other basic estimates (e.g. regression coefficient) AND variation (e.g. standard deviation) or associated estimates of uncertainty (e.g. confidence intervals)
[ ]	[x]	For null hypothesis testing, the test statistic (e.g. *F*, *t*, *r*) with confidence intervals, effect sizes, degrees of freedom and *P* value noted<br>*Give P values as exact values whenever suitable.*
[x]	[ ]	For Bayesian analysis, information on the choice of priors and Markov chain Monte Carlo settings
[x]	[ ]	For hierarchical and complex designs, identification of the appropriate level for tests and full reporting of outcomes
[ ]	[x]	Estimates of effect sizes (e.g. Cohen's *d*, Pearson's *r*), indicating how they were calculated

Our web collection on statistics for biologists contains articles on many of the points above.

Software and code

Policy information about availability of computer code

Data collection	ViiA 7 Real-time PCR system (Applied Biosystems), LSR Fortessa X20 (BD Biosciences), LSR Fortessa (BD Biosciences), Leica SP8
Data analysis	Image J ver1.52a, FlowJo V10, Graphpad Prism 8, Perseus 1.6

For manuscripts utilizing custom algorithms or software that are central to the research but not yet described in published literature, software must be made available to editors and reviewers. We strongly encourage code deposition in a community repository (e.g. GitHub). See the Nature Portfolio guidelines for submitting code & software for further information.

Data

Policy information about availability of data

All manuscripts must include a data availability statement. This statement should provide the following information, where applicable:
- Accession codes, unique identifiers, or web links for publicly available datasets
- A description of any restrictions on data availability
- For clinical datasets or third party data, please ensure that the statement adheres to our policy

All data associated with this study are present in the paper or the Supplementary Information. Source data are provided with this paper. The proteomics data used in this study are available in the Pride database under accession code PXD038584 (http://www.ebi.ac.uk/pride).

Research involving human participants, their data, or biological material

Policy information about studies with human participants or human data. See also policy information about sex, gender (identity/presentation), and sexual orientation and race, ethnicity and racism.

Reporting on sex and gender	The findings apply to both sexes.<br>Gender were not considered in the study design.<br>Gender-based analyses have not been performed because our study focused on the changes of phenotypes and functions of mast cells in rheumatoid arthritis in regardless of genders.
Reporting on race, ethnicity, or other socially relevant groupings	All patients involved are chinese.
Population characteristics	We collected fresh sample from 21 patients including 10 OA and 11 RA patients.<br>The mean age of the OA patients was 70.1±3.35 years, 90% were female.<br>The mean age of the RA patients was 64.74±9.96 years, 90.91% were female.<br>All patients have been diagnosed as arthritis and treated before. The detailed clinical characteristics have been displayed in the supplementary table 1.
Recruitment	Patients with OA and RA have been diagnosed before.<br>Patients with other autoimmune diseases, infectious diseases, or cancer were excluded.<br>We recruited patients with OA and RA undergoing artificial joint replacement surgery. Patients were included in outpatient clinics at any time point of the disease and written informed consent was obtained from each patient.
Ethics oversight	Experiments were approved by the Ethics Committee of Shanghai Sixth People's Hospital Affiliated to Shanghai Jiao Tong University School of Medicine-approval 2021-013, and written informed consent was obtained prior to participation.

Note that full information on the approval of the study protocol must also be provided in the manuscript.

Field-specific reporting

Please select the one below that is the best fit for your research. If you are not sure, read the appropriate sections before making your selection.

- [x] Life sciences
- [ ] Behavioural & social sciences
- [ ] Ecological, evolutionary & environmental sciences

For a reference copy of the document with all sections, see nature.com/documents/nr-reporting-summary-flat.pdf

Life sciences study design

All studies must disclose on these points even when the disclosure is negative.

Sample size	Sample size used determined based on previous experience and similar assays (PMID: 37060808, 33436641). No statistical test was used to pre-determine the sample sizes.
Data exclusions	No data was excluded.
Replication	Each in vitro experiment was run in technical duplicate, and the biological experiments were performed in independent replicates.<br>All experiments were repeated at least twice and up to 3 times, to ensure reproducibility of the results.<br>There were no experiments that we could not replicate independently in this study.
Randomization	In all experiments, mice were randomly assigned to each group and the order in which analysis procedures were performed were done at random.
Blinding	Blinding was not applied to almost experiments, because data collection and analysis were performed by the same investigators.

Reporting for specific materials, systems and methods

We require information from authors about some types of materials, experimental systems and methods used in many studies. Here, indicate whether each material, system or method listed is relevant to your study. If you are not sure if a list item applies to your research, read the appropriate section before selecting a response.

Materials & experimental systems

n/a	Involved in the study	
	x	Antibodies
	x	Eukaryotic cell lines
x		Palaeontology and archaeology
	x	Animals and other organisms
	x	Clinical data
x		Dual use research of concern
x		Plants

Methods

n/a	Involved in the study	
x		ChIP-seq
	x	Flow cytometry
x		MRI-based neuroimaging

Antibodies

Antibodies used

Immunohistochemistry antibodies:
anti-mast cell tryptase (Abcam, ab2378, [AA1], 1:500)
anti-Ki67 (Abcam, ab92742, [EPR3610], 1:250)
anti-CD3 (Abcam, ab16669, [SP7], 1:200)
anti-HLA-DR (Abcam, ab92511, [EPR3692], 1:250)
anti-CD3 (Abcam, ab11089, [CD3-12], 1:100)
anti-CD20 (Abcam, ab78237, [EP459Y], 1:100)
Anti-mouse IgG (H+L), F(ab')2 Fragment (Alexa Fluor® 488 Conjugate)(CST, 4408,1:1000)
Anti-rabbit IgG (H+L), F(ab')2 Fragment (Alexa Fluor® 594 Conjugate)(CST, 8889,1:1000)
Donkey anti-Rat IgG (H+L) Highly Cross-Adsorbed Secondary Antibody, Alexa Fluor™ 488(Thermo Scientific, A21208, 1:400)
Anti-mouse IgG (H+L), F(ab')2 Fragment (Alexa Fluor® 647 Conjugate)(CST, 4410,1:1000)

Western blotting antibodies:
anti-Phospho-PLCγ1 (CST, 14008, [D6M9S], 1:1000)
anti-Phospho-NF-κB p65 (CST, 3033, [93H1], 1:1000)
anti-Phospho-Akt (CST, 4060, [D9E], 1:1000)
Phospho-SAPK/JNK (CST, 4668, [81E11], 1:1000)
anti-Phospho-Erk1/2 (CST, 4370, [D13.14.4E], 1:1000)
anti-Phospho-p38 (CST, 4511, [D3F9], 1:1000)
anti-β-Actin (CST, 4970, [13E5], 1:1000)
anti-α-Tubulin (CST, 2144, 1:1000)
Anti-GPCR MRGX2 (Abcam, ab237047, 1:1000)
HRP-linked anti-rabbit-IgG antibody (CST, 7074, 1:2000)

Flow antibodies:
FITC anti-human CD45 (BioLegend, 368508, [2D1], 1:100)
APC anti-human CD117 (BioLegend, 313206, [104D2], 1:100)
PE anti-human FceRI (BioLegend, 334610, [AER-37], 1:50)
PerCP/Cy5.5 anti-human Ki67 (BD Biosciences, 561284, [B56], 1:100)
PerCP/Cy5.5 anti-human CD34 (BioLegend, 343521, [581], 1:100)
PE anti-human CD63 (Thermo Scientific, 12-0639-42, [H5C6], 1:100)
FITC anti-human HLA-DR, DP, DQ (BioLegend, 361705, [Tü39], 1:100)
PE/Cy7 anti-human CD40 (BioLegend, 313011, [HB14], 1:100)
PerCP/Cy5.5 anti-human CD80 (BioLegend, 305231, [2D10], 1:100)
PE anti-human OX40L (BioLegend, 326307, [11C3.1], 1:100)
BV421 anti-mouse CD3 (BioLegend, 100228, [17A2], 1:100)
FITC anti-mouse CD4 (Thermo Scientific, 11-0041-85, [GK1.5], 1:100)
PE/Cy7 anti-mouse IFN-γ (Thermo Scientific, 25-7311-82, [XMG1.2], 1:100)
PE anti-mouse IL-17 (BD Biosciences, 559502, [TC11-18H10], 1:100)
PE anti-mouse Foxp3 (Thermo Scientific, 12-5773-82, [FJK-16s], 1:50)
APC anti-mouse CD4 (BioLegend, 100411, [GK1.5], 1:100)
PE-Cyanine7 anti-mouse CD45.1 (Thermo Scientific, 25-0453-82, [A20], 1:100)
FITC anti-mouse CD45.2 (Thermo Scientific, 11-0454-82, [104], 1:100)
APC anti-mouse CD117 (Thermo Scientific, 17-1171-83, [2B8], 1:100)
PE anti-mouse FceRI (BioLegend, 134308, [MAR-1], 1:50)

Validation

All antibodies used in this study have been previously validated by the manufacturer, as stated on their associated product websites, and by our own lab in previous experiments.

Eukaryotic cell lines

Policy information about cell lines and Sex and Gender in Research

Cell line source(s)	LAD2 cells (NIH, NIAID#2021-0660)
Authentication	None of the cell lines used were authenticated.
Mycoplasma contamination	The cell lines were not tested for Mycoplasma contamination.
Commonly misidentified lines (See ICLAC register)	We did not use commonly misidentified lines.

Animals and other research organisms

Policy information about studies involving animals; ARRIVE guidelines recommended for reporting animal research, and Sex and Gender in Research

Laboratory animals	DBA/1J mice (8- to 10-week-old); C57BL/6 mice (8- to 10-week-old); CD45.1+ C57BL/6 mice (8- to 10-week-old). Mice were housed under 12 hour light/dark cycle at 20-24°C and 45-65% humidity.
Wild animals	No wild animals were used in the study.
Reporting on sex	We only used male mouse in order to successfully induce arthritis in mice.
Field-collected samples	No field collected samples were used in the study.
Ethics oversight	All animal care procedures were performed according to the National Institute of Health Guide for the Care and Use of Laboratory Animals. All animal experiments were conducted in accordance with the guidelines and approval of the Ethical Committee of the Shanghai Jiao Tong University School of Medicine-approval A-2019-064.

Note that full information on the approval of the study protocol must also be provided in the manuscript.

Clinical data

Policy information about clinical studies

All manuscripts should comply with the ICMJEguidelines for publication of clinical research and a completedCONSORT checklist must be included with all submissions.

Clinical trial registration	Ethics Committee of Shanghai Sixth People's Hospital Affiliated to Shanghai Jiao Tong University School of Medicine-approval 2021-013
Study protocol	The full trial protocol can be accessed on Chinese Clinical Trial Registry by number ChiCTR2000040167.
Data collection	Fresh synovial tissues and peripheral blood were obtained from patients with OA and RA undergoing artificial joint replacement surgery in Shanghai Sixth People's Hospital Affiliated to Shanghai Jiao Tong University School of Medicine. Patients with other autoimmune diseases, infectious diseases, or cancer were excluded.
Outcomes	Our study is a retrospective study of collecting clinical samples and clinical data without pre-definition of outcome measures.

Plants

Seed stocks	*Report on the source of all seed stocks or other plant material used. If applicable, state the seed stock centre and catalogue number. If plant specimens were collected from the field, describe the collection location, date and sampling procedures.*
Novel plant genotypes	*Describe the methods by which all novel plant genotypes were produced. This includes those generated by transgenic approaches, gene editing, chemical/radiation-based mutagenesis and hybridization. For transgenic lines, describe the transformation method, the number of independent lines analyzed and the generation upon which experiments were performed. For gene-edited lines, describe the editor used, the endogenous sequence targeted for editing, the targeting guide RNA sequence (if applicable) and how the editor was applied.*
Authentication	*Describe any authentication procedures for each seed stock used or novel genotype generated. Describe any experiments used to assess the effect of a mutation and, where applicable, how potential secondary effects (e.g. second site T-DNA insertions, mosiacism, off-target gene editing) were examined.*

Flow Cytometry

Plots

Confirm that:

☐ The axis labels state the marker and fluorochrome used (e.g. CD4-FITC).

☒ The axis scales are clearly visible. Include numbers along axes only for bottom left plot of group (a 'group' is an analysis of identical markers).

☒ All plots are contour plots with outliers or pseudocolor plots.

☒ A numerical value for number of cells or percentage (with statistics) is provided.

Methodology

Sample preparation	Whole blood was collected and peripheral blood mononuclear cells (PBMCs) were isolated using density gradient medium (StemCell). Human synovial tissues were harvested, mechanically dissociated and treated with RPMI 1640 medium (Thermo Scientific) containing 1 mg/mL collagenase 1 (Sigma), and 0.1 mg/mL DNase I (Roche) for 1h at 37°C.<br>Mouse ankle joints were harvested, and incubated in RPMI 1640 medium (Thermo Scientific) containing 1 mg/mL collagenase D (Sigma) for 1h at 37°C. Spleen cells were filtered through a 70 µm cell strainer. Where required, samples were treated with ACK buffer (150 mM NH3Cl, 1 mM KHCO3 and 1 mM EDTA) to lyse red blood cells. Subsequently, single cell suspensions were stained with viability marker (fixable viability dye eFluor780, Thermo Scientific) and primary fluorescently labelled antibodies of interest.
Instrument	LSR Fortessa X20 (BD Biosciences), LSR Fortessa (BD Biosciences)
Software	FlowJo V10
Cell population abundance	Cell population abondance were dependant of the organ analysed. In vitro spleen cells purity was ~95% post sorting.
Gating strategy	For all gating strategy in this article, we started gating with debris exclusion(appropriate FCS/SSC), removal of dead cells (negativity for viability marker) and singlets (FSC-A/FSC-H).<br>For mast cells in the human synovial tissues, we then gated as CD45+CD117+FcεRI+ cells.<br>For mast cell progenitors in the blood, we then gated as Lin-CD34hiCD117int/hiFcεRI+ cells<br>For CD4+T cells in the spleen of mice, we then gated as CD3+CD4+ cells and further subdivided into IFN-γ+, IL17-A+ and Foxp3+ cells.

☒ Tick this box to confirm that a figure exemplifying the gating strategy is provided in the Supplementary Information.
